# Plasticizers and prostate cancer: unraveling the link through network toxicology and machine learning

**DOI:** 10.3389/fonc.2026.1768691

**Published:** 2026-07-09

**Authors:** Yiting Jiang, Jiang Shi, Shiwang Yuan, Jun Qiao, Yuan Tian, Peng Chen, Qifang Zhang, Quliang Zhong, Tao Li, Guodong Yu

**Affiliations:** 1 Department of Otorhinolaryngology, The Affiliated Hospital of Guizhou Medical University, Guiyang, China; 2 Department of Urology, The Affiliated Hospital of Guizhou Medical University, Guiyang, China; 3 Guizhou Medical University, Guiyang, China

**Keywords:** machine learning algorithms, network toxicology, plasticizers, PLK1, prostate cancer

## Abstract

**Background:**

Plasticizers, as widespread environmental endocrine disruptors, are increasingly linked to an elevated risk of prostate cancer (PCa). However, the specific molecular mechanisms by which they drive PCa initiation and progression remain incompletely elucidated. Addressing this knowledge gap is crucial for assessing environmental health risks and identifying potential intervention targets.

**Methods:**

This study employed a multi-level integrated research strategy. First, the toxicological profiles of target plasticizers were predicted using ADMETlab and ProTox platforms. Second, plasticizer-related targets were identified by integrating multiple databases and then cross-referenced with differentially expressed genes in PCa from TCGA and GEO cohorts to obtain shared targets. Subsequently, a protein-protein interaction (PPI) network was constructed and analyzed topologically. GO and KEGG enrichment analyses were performed to explore underlying biological processes and pathways. A total of 98 combination prediction models based on 10 machine learning algorithms were developed and evaluated to identify core prognostic genes. Furthermore, single-cell and spatial transcriptomics data were utilized to examine the expression localization of core genes within the tumor microenvironment. Molecular docking simulations were conducted to validate the binding affinity between plasticizers and core target proteins. Finally, *in vitro* experiments demonstrated the pro-tumorigenic effects of DMP and its regulatory role in PLK1 expression in prostate cancer cells.

**Results:**

Toxicity predictions confirmed the carcinogenic potential of DEP, DMP, and DOP. A total of 183 bridging genes connecting plasticizers and PCa were identified. Enrichment analysis revealed their significant involvement in key pathways including inflammatory response, cell cycle, p53 signaling, and chemical carcinogenesis. PPI network analysis preliminarily screened hub genes such as ALB and MMP9. Through systematic machine learning modeling and prognostic analysis, the core targets were further narrowed down to PLK1, ALB, and CCNA2. Among these, high expression of PLK1 was significantly associated with shorter disease-free survival in multiple independent cohorts. Molecular docking results indicated that all three plasticizers could bind stably to the PLK1 protein with high affinity (binding free energy < -5.0 kcal/mol). Single-cell and spatial transcriptomic analyses showed high expression of PLK1 in tumor epithelial cells. *In vitro* experiments confirmed that DMP promotes the proliferation, migration, and invasion of PCa cells, as well as upregulates PLK1 expression. Pan-cancer analysis further indicated that PLK1 is commonly overexpressed in various cancers and associated with poor prognosis.

**Conclusion:**

This study integrates computational toxicology, bioinformatics, machine learning, and experimental validation to reveal that common plasticizer exposure may promote PCa progression through dysregulation of cell cycle and inflammatory pathways, with PLK1 identified as a central molecular target. These findings establish a multi-omics evidence chain supporting the carcinogenic potential of environmental endocrine disruptors and provide a scientific basis for considering PLK1 as both a biomarker for risk assessment and a therapeutic target in plasticizer-associated PCa.

## Background

Plasticizers are chemical additives used to enhance the flexibility and processability of plastics. Common types include diethyl phthalate (DEP), dimethyl phthalate (DMP), and dioctyl phthalate (DOP). They are ubiquitous in daily chemicals, plastic toys, medical devices, and food packaging, making human exposure nearly unavoidable (1–3). These compounds are characterized by their lipophilicity and environmental persistence, leading to bioaccumulation in the environment and organisms (4, 5). More concerning is their structural resemblance to endogenous hormones, which allows them to interfere with endocrine function by mimicking or antagonizing hormonal action. Consequently, increasing research focuses on their potential role in promoting hormone-related cancers, such as prostate cancer (PCa) (5–7).

PCa is a malignant tumor with high clinical heterogeneity, presenting as either an aggressive form prone to rapid progression and metastasis or an indolent form that may remain asymptomatic and slow-growing for years. As the most frequently diagnosed cancer and the second leading cause of cancer-related death among men worldwide, PCa poses a major public health threat and imposes a substantial medical burden (8, 9). However, the exact mechanisms underlying its high incidence remain incompletely understood. Established risk factors include age, ethnicity, and family history, while the roles of environmental exposures, lifestyle, and dietary components are less defined (8, 10). Emerging evidence suggests that environmental endocrine disruptors, represented by plasticizers, may play a key role in PCa development (6, 7, 11–13). Nonetheless, current research in this area has limitations: (i) epidemiological studies often rely on serum biomarkers and lack direct histopathological correlation; (ii) preclinical experiments frequently employ high-dose exposure models that do not reflect realistic human exposure levels; and (iii) the specific molecular mechanisms by which plasticizers influence PCa initiation and progression have not been fully elucidated. Therefore, the precise association and mechanistic links between environmental plasticizers and PCa await more in-depth investigation.

To address these knowledge gaps, this study was designed with the following objectives: (1) to identify common targets shared by DEP, DMP, and DOP exposure and prostate cancer by integrating toxicogenomic databases with transcriptomic data from TCGA and GEO cohorts; (2) to construct and validate a robust prognostic model based on these targets using 10 machine learning algorithms across 98 algorithm combinations; and (3) to experimentally validate the functional role of the core target PLK1 in mediating plasticizer−induced malignant phenotypes in prostate cancer cells through *in vitro* assays, including proliferation, migration, invasion, and PLK1 knockdown experiments. By combining network toxicology, multi−omics analysis, and experimental validation, this study aims to uncover the molecular mechanisms linking common plasticizer exposure to PCa progression and to evaluate PLK1 as a potential biomarker and therapeutic target for plasticizer−associated prostate cancer.

## Materials and methods

### Data sources

This study integrated transcriptomic data and matched clinical information from 1,028 PCa patients. The data were sourced from two components: (1) the TCGA-PRAD cohort (accessed via the UCSC Xena platform), and (2) three independent cohorts from the GEO database (GSE21032, GSE70770, and GSE116918). To enhance statistical power, GSE21032 and GSE70770 were merged into a combined cohort, with technical batch effects corrected using the ComBat algorithm from the ‘sva’ package. To ensure data reliability, patients lacking disease recurrence information or with follow-up times of less than one month were excluded. Additionally, a single-cell transcriptomic dataset (GSE185344) and spatial transcriptomic data from one PCa patient (GSM7841733) from the GEO database were also incorporated.

### Toxicity prediction

Given their widespread environmental presence, DEP, DMP, and DOP were selected for subsequent analysis. Using their canonical SMILES structures retrieved from PubChem, the toxicity profiles and related parameters of these compounds were systematically predicted via the ProTox 3.0 and ADMETlab 3.0 platforms. These computational tools cover multiple critical toxicological endpoints, enabling a comprehensive assessment of absorption, distribution, metabolism, excretion, and toxicity (ADMET) properties. This provides an important theoretical foundation for subsequent experimental validation and risk assessment (14, 15).

### Identification of plasticizer targets

The canonical chemical structures and SMILES notations of DEP, DMP, and DOP were retrieved from PubChem and submitted for target prediction analysis to three authoritative databases: SwissTargetPrediction, TargetNet, and PharmMapper. An initial prediction was performed using SwissTargetPrediction and TargetNet, followed by further target identification via the PharmMapper platform. During analysis, corresponding thresholds were applied to filter for high-confidence human targets with a prediction probability greater than zero. Finally, results from all three databases were integrated, and gene name standardization was performed using UniProt to eliminate redundant entries.

### Identification of PCa targets

We analyzed transcriptomic data from 534 patient tissue samples in the TCGA-PRAD cohort, comprising 483 tumor tissues and 51 adjacent normal tissues. Differential expression analysis was performed using the DESeq2 R package with a relatively lenient threshold of |log_2_FoldChange| > 0.50 and adjusted p-value < 0.05. This approach was intended to avoid missing genes that may exert effects through cumulative contributions in key pathways. The significantly differentially expressed genes identified were considered potential therapeutic targets for prostate cancer (PCa) and were used for subsequent in-depth studies.

### Plasticizer-PCa target identification and PPI network construction

To identify common targets between plasticizers and PCa, a Venn analysis was first conducted. Subsequently, protein-protein interaction (PPI) analysis was performed on the overlapping targets using the STRING database (confidence score threshold set to ≥ 0.4). This database integrates known and predicted protein associations, including physical interactions and functional linkages. After obtaining the PPI data, non-essential targets were filtered out to construct a high-quality interaction network. The network was imported in TSV format into Cytoscape 3.9.0 for visualization and topological analysis. Using the built-in “Centiscape 2.0” tool, multiple topological parameters—including degree centrality, closeness centrality, and betweenness centrality—were calculated to evaluate the importance of each node within the network. Genes were ultimately ranked by their degree centrality value, with a higher value indicating a more pivotal hub status.

### Enrichment analysis

Gene Ontology (GO) systematically categorizes gene functions into three classes: Cellular Component (CC), Molecular Function (MF), and Biological Process (BP). The Kyoto Encyclopedia of Genes and Genomes (KEGG) links genomic information with functional pathways at a systems level. GO and KEGG functional enrichment analyses of the plasticizer-PCa targets were performed using the R package ‘clusterProfiler’.

### Development and validation of a plasticizer-PCa risk model

Following previously established modeling workflows (16, 17), a systematic modeling approach based on 10 machine learning algorithms was used to construct 98 combination models. The aim was to establish a high-accuracy diagnostic model and identify core genes closely associated with the disease. The TCGA-PRAD cohort served as the training set, while the combined GSE cohort and the GSE116918 dataset served as external validation sets. Model performance was ranked by calculating the average C-index, and the best-performing combination was selected as the final diagnostic model. Patients in the TCGA-PRAD and validation cohorts were stratified into high- and low-risk groups based on the median risk score. Prognostic differences between groups were analyzed using Kaplan-Meier survival curves, and the model’s classification efficacy was evaluated via the area under the receiver operating characteristic curve (AUC).

### Single-cell and spatial transcriptomic analysis

For single-cell RNA sequencing analysis, stringent quality control was applied: only cells with mitochondrial gene content below 20%, expressing more than 200 genes, and detected in at least three cells were retained. A total of 36,424 high-quality cells were included for subsequent analysis. Data were log-normalized, and the top 2000 highly variable genes were identified using the FindVariableFeatures function. Dimensionality reduction was performed via principal component analysis, followed by batch effect correction using the Harmony algorithm. Subsequently, soft k-means clustering was applied, and cell clustering was finalized using the FindClusters function at a resolution of 0.3. Cell type annotation was based on classical marker genes, differential expression patterns, and known cellular lineage characteristics. Spatial transcriptomic data were also preprocessed, normalized, and standardized using the Seurat R package, with tissue region annotation performed according to classical marker genes. Further analysis of the expression distribution of key plasticizer-PCa target genes across different cell types aimed to reveal potential cellular mechanisms linking plasticizer exposure to PCa progression.

### Molecular docking

To elucidate the interaction mechanisms between plasticizers and core target proteins, molecular docking simulations were performed. The molecular structures of DEP, DMP, and DOP were obtained from PubChem, and the three-dimensional structures of the target proteins were sourced from the AlphaFold Protein Structure Database. AutoDockTools 1.5.7 was used to prepare the molecular and protein structures and to perform docking simulations, predicting binding modes, binding affinity (expressed as binding free energy ΔG), and potential functional impacts. A binding free energy below 0 kcal/mol indicates spontaneous binding, while values below -5.0 kcal/mol suggest a stable complex with strong binding capability.

### Cell culture and processing

The human prostate epithelial cell line (RWPE-1) and prostate cancer cell lines (DU145 and PC-3) were obtained from the Cell Bank of the Chinese Academy of Sciences. Cells were maintained at 37 °C in a 5% CO_2_ atmosphere using culture conditions specified by the supplier. To evaluate the effect of DMP on PCa cells, we prepared and administered it at a concentration of 5 μM based on previous literature. For the PLK1 knockdown experiments in DU145 and PC-3 cells, transfection was performed using Lipofectamine 8000 transfection reagent and Opti-MEM medium, strictly following the manufacturer’s instructions. The siRNA targeting PLK1 was synthesized by Sangon Biotech Co., Ltd.

### RNA isolation and quantitative real-time PCR

Gene expression validation was performed using standard molecular biology techniques. Total RNA was extracted from cells using TRIzol reagent, and sample purity was assessed with a NanoDrop 2000 spectrophotometer (A260/A280 ratio > 1.8 was considered acceptable). Qualified RNA was reverse-transcribed into cDNA using the PrimeScript™ RT Reagent Kit, followed by qPCR analysis on a QuantStudio 5 system using the Premix Ex Taq™ Kit. Reaction conditions were set as follows: pre-denaturation at 95 °C for 30 seconds, followed by 40 cycles of 95 °C for 5 seconds and 60 °C for 34 seconds. All experiments were performed with three technical replicates. Primer sequences used are as follows: F: GCACAGTGTCAATGCCTCCAAG; R: GCCGTACTTGTCCGAATAGTCC.

### Western blotting

Total protein was extracted from DU145 and RWPE-1 cells using RIPA lysis buffer (Solarbio, China) supplemented with a protease inhibitor cocktail (Yeasen, China). Protein samples were separated by 10% SDS-polyacrylamide gel electrophoresis and then transferred onto PVDF membranes using a wet transfer method. The membranes were blocked with 5% skim milk at room temperature for 1 hour, followed by overnight incubation at 4 °C with an anti-PLK1 primary antibody (1:1000 dilution; catalog #10305-1-AP, Proteintech). After washing with TBST, the membranes were incubated with the corresponding species-specific secondary antibody at room temperature for 2 hours.

### EdU incorporation assay

The effect of DMP on prostate cancer cell proliferation was assessed using the 5-ethynyl-2′-deoxyuridine (EdU) assay kit (Beyotime, Shanghai, China). DU145 and PC-3 cells were seeded in 24-well plates at a density of 3×10³ cells per well and allowed to adhere overnight. Cells were then exposed to DMP (5μM) with or without PLK1 knockdown for 24h. EdU working solution (20μL) was added to each well and incubated for 2h at 37°C. After fixation and staining according to the manufacturer’s instructions, EdU-positive nuclei were visualized under a fluorescence microscope. Proliferation rates were quantified by counting positively stained cells in at least five randomly selected fields per well.

### Wound healing migration assay

Cell migratory capacity was evaluated by a wound healing assay. DU145 and PC-3 cells were cultured in 6-well plates until they formed a confluent monolayer. A linear scratch was introduced across the monolayer using a sterile 200-μL pipette tip, and cellular debris was removed by gentle washing with PBS. To minimize the influence of proliferation on migration, the medium was replaced with low-serum (1% FBS) culture medium. Wound closure was observed under a phase-contrast microscope at 0h and 48h after scratching. Migration ability was expressed as the percentage of wound area reduction relative to the initial scratch width, measured using ImageJ software.

### Transwell invasion assay

Invasion potential was examined using Transwell chambers (8-μm pore size; Corning, USA). Treated and control cells were harvested, resuspended in serum-free RPMI-1640 medium, and seeded into the upper chambers at a density of 5×10^4^ cells per well. The lower chambers were filled with RPMI-1640 containing 10% FBS as a chemoattractant. After 24h of incubation at 37°C, non-invading cells on the upper surface of the membrane were removed with a cotton swab. Cells that had migrated to the lower surface were fixed with 4% paraformaldehyde, stained with 0.1% crystal violet, and photographed under an inverted microscope. For each replicate, five random fields were captured, and the number of invaded cells was counted.

### Statistical analysis

For RNA-seq data, differential expression analysis was performed using DESeq2, which models count data based on the negative binomial distribution and does not assume normality or homogeneity of variance. To control for multiple testing, the Benjamini-Hochberg method was applied to maintain the false discovery rate below 5%. For *in vitro* experiments, normality was assessed using the Shapiro–Wilk test; when the normality assumption was met, parametric tests (paired or unpaired Student’s t-test) were used; otherwise, non-parametric tests (Wilcoxon signed-rank test or Mann–Whitney U test) were applied. Homogeneity of variance was evaluated using Levene’s test, and Welch’s correction was applied when necessary. All statistical tests were two-sided, and p < 0.05 was considered statistically significant.

## Results

### Preliminary analysis of the plasticizer toxicity network

Computational analysis using ADMETlab 3.0 and ProTox 3.0 revealed that DEP, DMP, and DOP exhibit significant carcinogenic potential and bioaccumulative properties. Their pharmacokinetic profiles were characterized by high oral absorption (Caco-2 permeability Papp > 1.2×10^-6^ cm/s) and high lipophilicity (LogP > 5). Toxicity predictions further confirmed the carcinogenic and mutagenic potential of all three compounds. This computational toxicology evidence provides theoretical support for the hypothesis that plasticizers promote PCa initiation and progression, establishing a direction for subsequent mechanistic research.

### Identification of plasticizer-PCa targets

**Figure 1:**
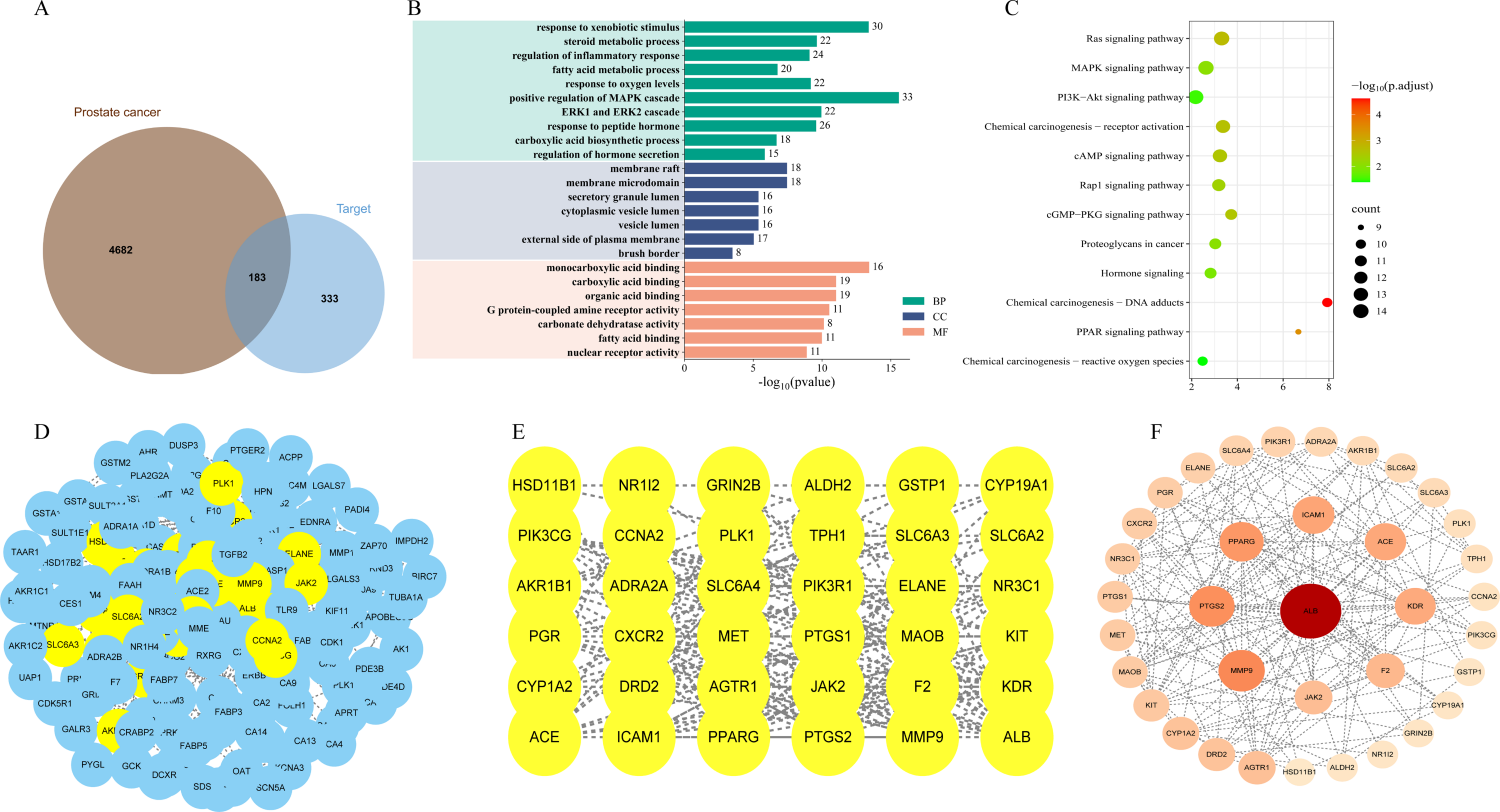
Identification, enrichment analysis, and PPI network construction of plasticizer-PCa targets. **(A)** Plasticizer-PCa overlapping gene; **(B)** GO enrichment analysis; **(C)** KEGG enrichment analysis; **(D-F)** Construction of PPI network and screening of core targets.

After integrating target prediction results from the aforementioned multiple databases and removing duplicates, 516 unique plasticizer-related target genes were identified. Differential expression analysis further identified 4,865 differentially expressed genes in tumor tissues. Venn analysis identified 183 overlapping genes associated with both plasticizers and PCa (**Figure 1A**). These genes may serve as potential therapeutic targets in plasticizer-mediated PCa pathogenesis.

### Enrichment analysis of plasticizer-PCa targets

GO functional enrichment analysis of the 183 plasticizer-PCa-related genes revealed significant enrichment in biological processes such as inflammatory and oxidative stress responses, hormone metabolism and secretion, metabolic reprogramming, and energy metabolism pathways (e.g., the MAPK signaling pathway) (**Figure 1B**). KEGG pathway analysis further indicated that these genes are involved in key pathways, including the PPAR signaling pathway, steroid hormone biosynthesis, MAPK signaling pathway, Ras signaling pathway, the PI3K-Akt signaling pathway, and other crucial oncogenic pathways, as well as chemical carcinogenesis (encompassing DNA adduct formation, receptor activation, and reactive oxygen species effects) (**Figure 1C**). In summary, plasticizers may promote the initiation and progression of PCa by interfering with inflammatory responses, cell proliferation and apoptosis, and multiple carcinogenic pathways.

### Construction of the PPI network for plasticizer-PCa targets

The 183 overlapping plasticizer-PCa target genes were imported into the STRING database for protein-protein interaction (PPI) analysis, with a confidence threshold set to ≥ 0.4. After removing disconnected nodes, the final network contained 176 target proteins. Cytoscape 3.10.3 was used to visualize the PPI network, with nodes arranged by degree; darker colors and larger diameters indicate stronger interactions within the network. Topological analysis identified five core targets within the plasticizer-PCa interaction network: ALB, MMP9, PTGS2, PPARG, and ICAM1 (**Figures 1D–F**). This network visualization not only clearly illustrates interactions among key targets but also provides important clues for further exploration of the potential molecular mechanisms linking plasticizers to PCa.

### Development and evaluation of the plasticizer-PCa prediction model

**Figure 2:**
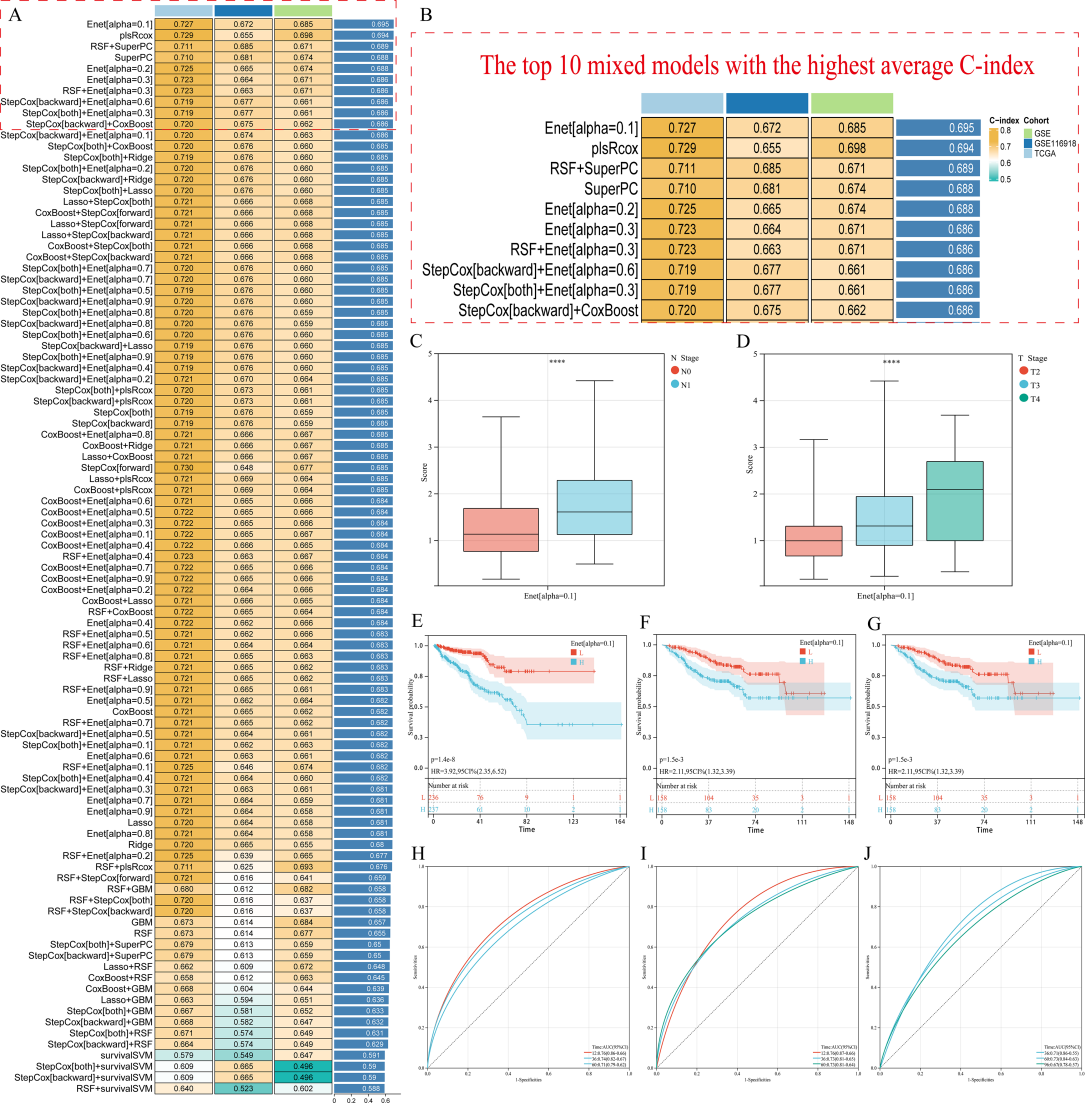
Establishment and validation of Plasticizer-PCa target related risk prediction model. **(A, B)** The average C-index of 101 robot algorithm combinations; **(C, D)** Risk Score Stratified by Clinical Stage; **(E-G)** Comparing survival outcomes using the risk score as a stratifier, **(E)** TCGA-PRAD, **(F)** GSE, GSE116918; **(H-J)** Performance of the risk score in identifying DFS patients by ROC analysis, **(H)** TCGA-PRAD, **(I)** GSE, **(I)** GSE116918.

Based on 18 plasticizer-PCa genes significantly associated with DFS identified by univariate Cox regression analysis (**Supplementary Figure 1**), a systematic evaluation of 98 algorithm combinations identified the Enet[alpha=0.1] model as the optimal predictive model. This model integrated 13 key genes (**Figures 2A, B**) and achieved an average C-index of 0.695. Clinicopathological analysis revealed that the model score was significantly associated with higher pathological stage (**Figures 2C, D**). Kaplan-Meier analysis further confirmed that high-risk patients exhibited worse clinical outcomes in three independent cohorts: TCGA-PRAD (**Figure 2E**), the combined GSE cohort (**Figure 2F**), and GSE116918 (**Figure 2G**). Receiver operating characteristic (ROC) curves validated the model’s predictive efficacy for disease progression at different time points. In the TCGA-PRAD cohort, the area under the curve (AUC) values for 1, 3, and 5 years were 0.76, 0.74, and 0.71, respectively (**Figure 2H**); in the combined GSE cohort, they were 0.76, 0.73, and 0.73, respectively (**Figure 2I**); and in the GSE116918 cohort, the AUC values for 3, 5, and 8 years were 0.71, 0.73, and 0.67, respectively (**Figure 2J**). These results collectively demonstrate the important prognostic value of the risk model constructed from 13 plasticizer-PCa genes in PCa, suggesting these genes may play crucial roles in plasticizer-induced PCa development.

### Determination of core plasticizer-PCa targets

**Figure 3:**
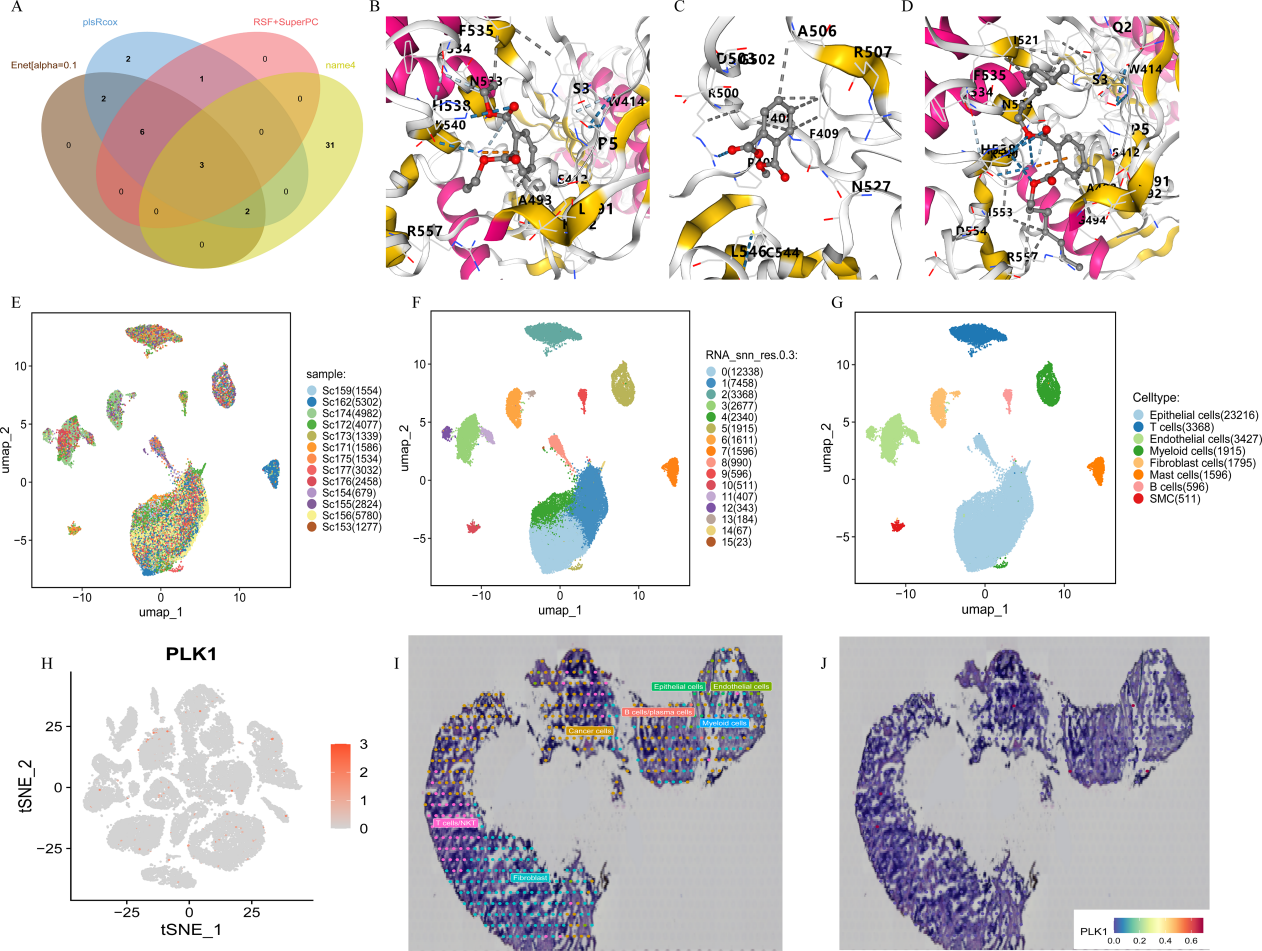
The effect of plasticizers on PIK1 and the expression localization of PLK1. **(A)** Identification of core genes; **(B-D)** Molecular docking between plasticizers and PLK1 protein, **(B)** DEP, **(C)** DMP, **(D)** DOP; **(E-G)** Single cell correction and grouping situation; **(H)** Expression of PLK1 in PCa microenvironment; **(I)** Localization of microenvironment space in prostate cancer; **(J)** Spatial localization of PLK1 in the microenvironment of prostate cancer.

**Figure 4:**
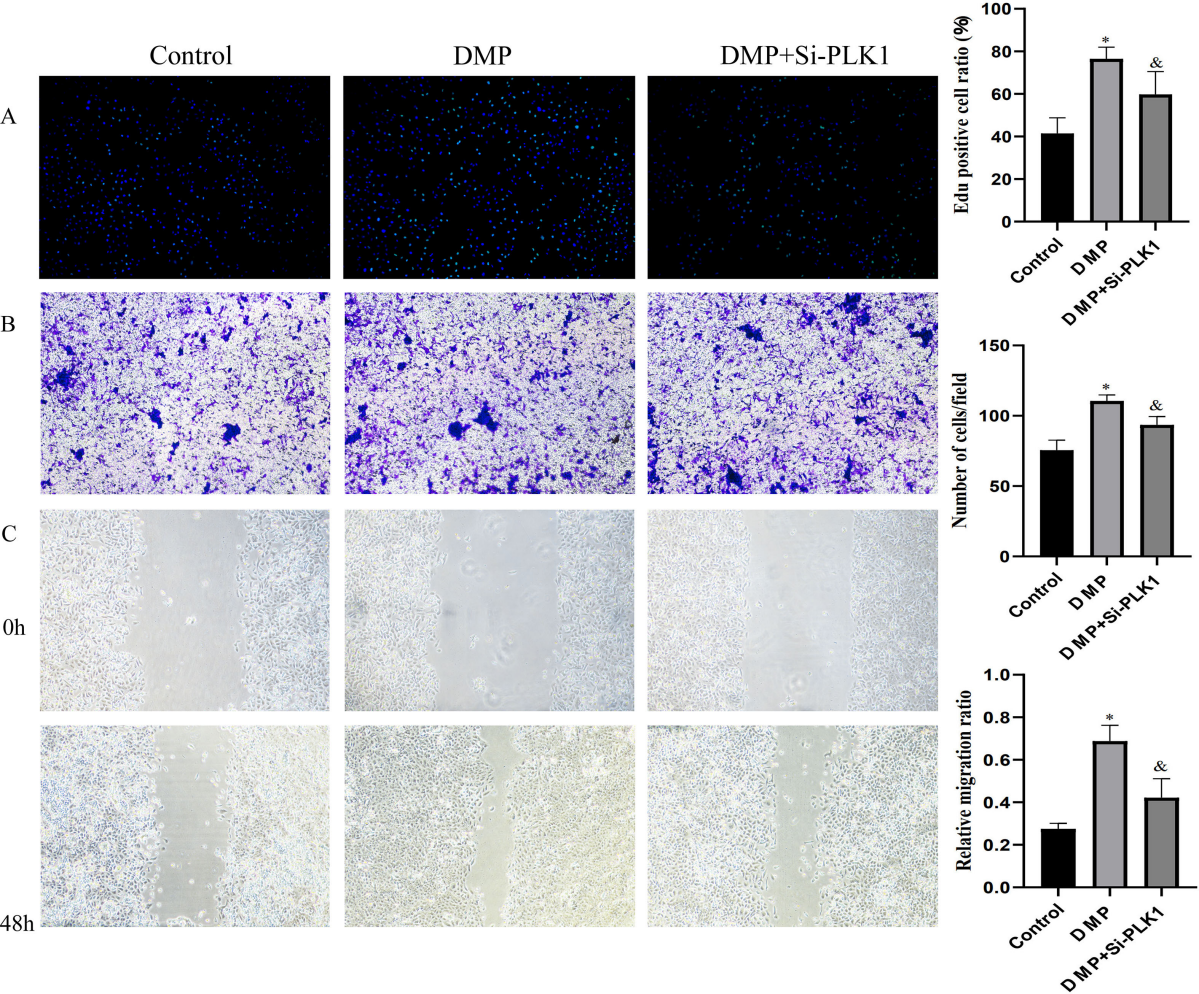
The effect of DMP on DU145 cells in vitro. **(A)** EdU proliferation assay; **(B)** Transwell invasion assay; **(C)** Wound healing migration assay. Data are presented as mean ± SD from three independent experiments. *p < 0.05 vs. Control; &p < 0.05 vs. DMP.

To further pinpoint core genes, Venn analysis identified three candidates: ALB, CCNA2, and PLK1 (**Figure 3A**). These genes were not only present within the top three hybrid models with the optimal C-index among the 98 algorithm combinations but also represented hub genes with key topological significance in the PPI network. Subsequent Kaplan-Meier survival analysis revealed that, among these three genes, only PLK1 showed a significant association with DFS across all three cohorts, where its high expression corresponded to shorter DFS (**Supplementary Figure 2**). Further molecular docking results indicated that all three common plasticizers (DEP, DMP, and DOP) could bind stably to the PLK1 protein, with docking energies of -6.1 kcal/mol, -5.8 kcal/mol, and -6.5 kcal/mol, respectively (**Figures 4B–D**). These findings collectively suggest that PLK1 may play a central role in plasticizer-mediated prostate cancer pathogenesis, and it was therefore selected as the focus for subsequent investigation.

### Expression localization and validation of PLK1

**Figure 5:**
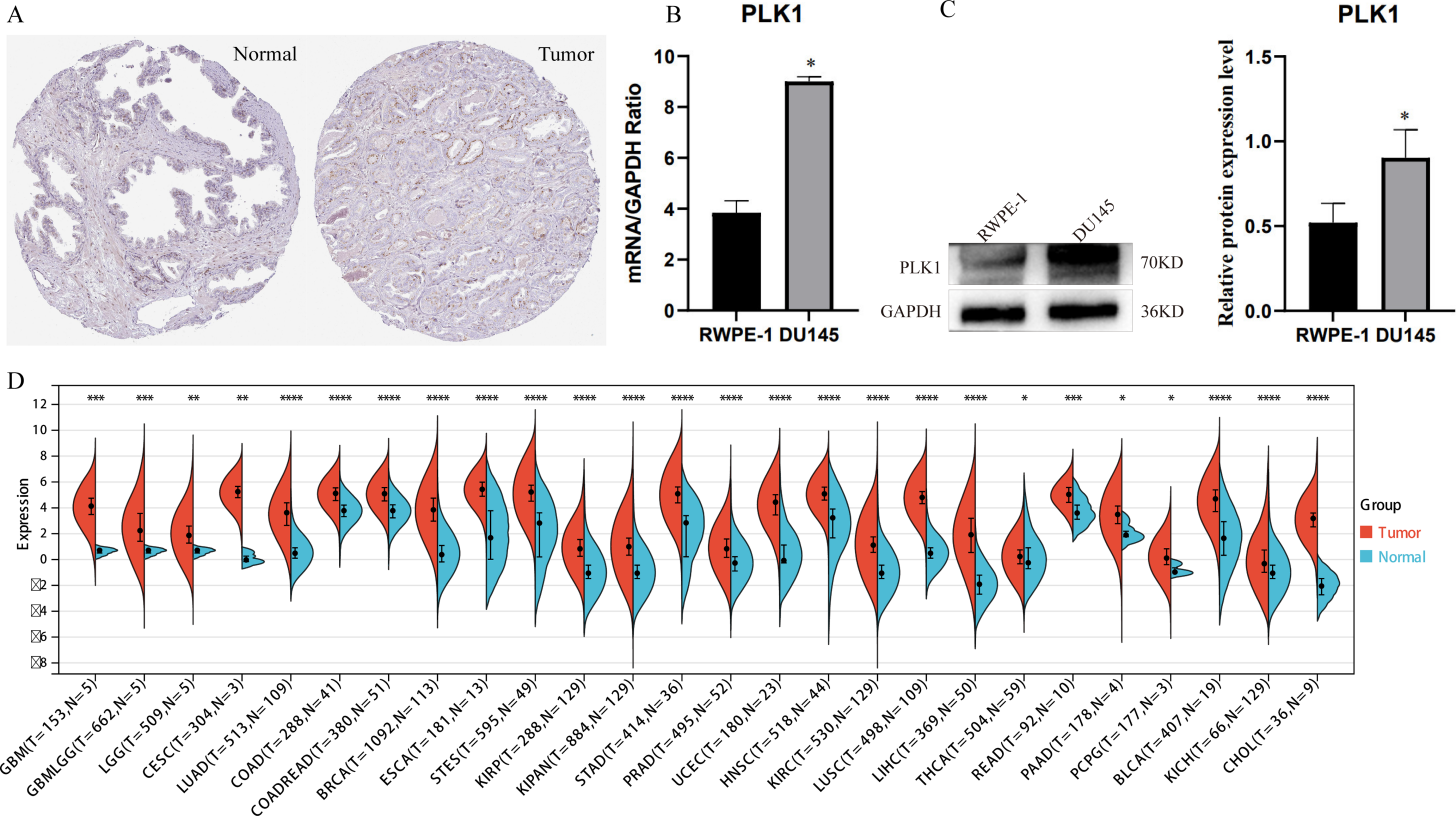
Expression validation of PLK1. **(A)** Protein expression of PLK1 in normal and prostate cancer tissues based on immunohistochemistry from the Human Protein Atlas (HPA) database. **(B)** Quantitative RT-PCR analysis showing PLK1 mRNA levels in normal prostate epithelial cells (RWPE-1) and prostate cancer cells (DU145). **(C)** Western blot analysis showing PLK1 protein expression in RWPE-1 and DU145 cells. **(D)** Pan cancer analysis results of PLK1. Data are presented as mean ± SD. *p < 0.05, p < 0.01, p < 0.001, ***p < 0.0001

After batch effect correction using the Harmony algorithm, cell distributions across samples converged, indicating effective control of batch effects (**Figure 3E**). At a resolution of 0.3, a total of 17 cell subclusters were identified (**Figure 3F**). Based on classical cell markers, these subclusters were further annotated into seven major cell types: epithelial cells, T cells, B cells, macrophages, endothelial cells, mast cells, and smooth muscle cells (**Figure 3G**). The core gene PLK1 was widely expressed across multiple cell types (**Figure 3H**). Spatial transcriptomic analysis further revealed its prominent expression, particularly within tumor cell regions (**Figures 3I, J**). Validation using the Human Protein Atlas (HPA) database showed moderate PLK1 expression levels in both cancerous and normal prostate tissues (**Figure 5A**). qPCR experiments confirmed that PLK1 mRNA levels were significantly elevated in the DU145 cell line (**Figure 5B**). Subsequent western blot (WB) analysis further demonstrated upregulation of PLK1 protein expression in DU145 cells (**Figure 5C**).

### Pan-cancer analysis of PLK1

Pan-cancer analysis (based on the SangerBox database) revealed that PLK1 was significantly upregulated in almost all cancer types (**Figure 5D**). Its high expression was significantly associated with poor progression-free survival in various tumors, including GBMLGG, BRCA, BLCA, PRAD, LGG, ACC, KICH, KIPAN, KIRP, KIRC, LIHC, PAAD, MESO, UCEC, LUAD, THCA, SKCM-M, SARC, and SKCM (**Supplementary Figure 3**). These results collectively underscore the important role of PLK1 in tumorigenesis and development, suggesting it may serve as a key molecular target in plasticizer-driven carcinogenesis.

### *In vitro* effects of DMP on PCa cells

Considering that the molecular docking results showed the most stable binding of DMP to PLK1, we selected DMP to validate our molecular docking and bioinformatics findings. Initially, qPCR and Western blot analysis revealed that DMP exposure increased PLK1 mRNA and protein levels in DU145 cells, while siRNA-mediated PLK1 knockdown effectively reduced its expression (**Supplementary Figure 4**). Subsequently, cellular functional assays demonstrated that DMP treatment significantly enhanced PCa cell proliferation (EdU assay, **Figure 4A**), invasion (Transwell assay, **Figure 4B**), and migration (wound healing assay, **Figure 4C**). Notably, inhibition of PLK1 expression reversed these pro-tumor effects of DMP (**Figures 4A–C**). In conclusion, these findings further emphasize that DMP may promote PCa malignancy by upregulating PLK1.

To further confirm the generalizability of these observations, we repeated the experiments in PC-3 cells. As shown in **Supplementary Figure 5**, DMP promoted similarly proliferation, invasion, and migration of PC-3 cells (**Supplementary Figures 5A–C**). Knockdown of PLK1 abolished these DMP-induced malignant phenotypes (**Supplementary Figures 5A–C**). These results indicate that the promoting effect of DMP on prostate cancer cells is not restricted to a single cell line and that PLK1 plays a central role in mediating plasticizer-induced tumor progression across different prostate cancer subtypes.

## Discussion

As exogenous environmental pollutants, plasticizers can disrupt physiological homeostasis by interfering with the endocrine and metabolic systems, exerting multiple adverse effects on the reproductive, nervous, and immune systems (1–3). With industrial development, plasticizers have become widespread environmental contaminants. Long-term, multi-pathway human exposure to these substances may pose significant health risks, particularly related to their carcinogenic properties (1–5). Multiple lines of laboratory evidence indicate a clear association between plasticizer exposure and an increased risk of PCa (18–23). Collectively, these studies, ranging from population data to cellular-level investigations, confirm a strong link between plasticizer exposure and elevated PCa risk.

Despite existing research confirming an association between plasticizer exposure and PCa risk, several key scientific questions in this field remain unresolved. Firstly, most current studies focus on epidemiological correlation analyses, with insufficient exploration of the specific molecular targets and mechanisms by which plasticizers drive PCa initiation and progression. Secondly, traditional experiments are often limited to detecting changes in specific protein expression, lacking a systematic analysis of overall biological processes and interaction networks. In contrast, high-throughput targeted screening methods can more comprehensively reveal molecular alterations induced by plasticizers. Furthermore, the prognostic value of plasticizer-related molecular markers in PCa patients remains unclear, limiting their clinical translational potential. These research gaps highlight the necessity of employing an integrated multi-omics strategy to systematically elucidate the mechanisms underlying the plasticizer-PCa association.

To address the existing research gaps, this study adopted a multidisciplinary strategy integrating multi-omics data to systematically investigate the association between plasticizer exposure and PCa. By combining bioinformatics analysis, machine learning modeling, molecular docking simulations, and *in vitro* experimental validation, we constructed a multi-level research framework aimed at systematically revealing the molecular mechanisms by which plasticizers influence PCa development. Based on environmental prevalence and human exposure risk, three common plasticizers (DEP, DMP, and DOP) were selected as the subjects of this study. Toxicity assessment confirmed the bioaccumulation potential and carcinogenic risk of these compounds. Subsequent bioinformatic analysis identified 183 bridging genes that may connect plasticizer exposure to PCa pathogenesis. KEGG and GO enrichment analyses suggested that plasticizers may promote the initiation and development of PCa through multiple pathways and targets by regulating biological processes such as inflammatory responses, oxidative stress, cell proliferation, and metabolic reprogramming, thereby interfering with key cancer-related signaling pathways including TP53, PPAR, and AMPK.

To further screen for key molecules, we established a robust computational framework integrating 98 combinations of 10 machine learning algorithms. Combined with survival analysis, this framework ultimately identified PLK1 as a core target within the plasticizer-PCa network, with its expression levels significantly correlated with patient prognosis across multiple independent cohorts. Although ALB and CCNA2 emerged as hub genes in the PPI network and initial models, ALB’s network centrality may reflect its role as a carrier protein rather than a direct oncogenic driver, while CCNA2 upregulation typically accompanies accelerated proliferation as a consequence rather than a specific driver of tumorigenesis. In contrast, PLK1 functions as an upstream kinase with clear druggable potential and mechanistic specificity. Single-cell and spatial transcriptomic analyses revealed its specific expression pattern within the tumor microenvironment, and pan-cancer analysis confirmed its overexpression across most malignancies, underscoring PLK1’s critical oncogenic role in plasticizer-associated PCa progression. For this reason, PLK1 was identified as the key molecule in this study.

As a serine/threonine kinase, PLK1 plays a central role in regulating the cell cycle, participating in key events such as mitotic entry, centrosome maturation, spindle assembly, sister chromatid cohesion, and cytokinesis (24, 25). This kinase is overexpressed in various malignancies and has been closely linked to tumorigenesis and progression, garnering increasing attention in cancer research (26–28). Particularly in PCa, previous studies have emphasized that PLK1 is significantly associated with disease progression, including metastasis, recurrence, and anti-androgen resistance (29–31), and is considered one of the primary mechanisms underlying anti-androgen therapy tolerance in PCa. Our study observed a similar trend: *PLK1* is highly expressed in most cancer types, including PCa, and its overexpression is significantly associated with shorter progression-free survival in patients with PCa and other tumors.

Our study observed a similar trend: PLK1 is highly expressed in most cancer types, including PCa, and its overexpression is significantly associated with shorter progression-free survival in patients with PCa and other tumors. Furthermore, our *in vitro* experiments demonstrated that DMP exposure upregulates PLK1 expression and enhances malignant behaviors in both DU145 and PC-3 cells, and that silencing PLK1 abrogates these effects. This consistency across two androgen-independent prostate cancer cell lines strengthens the conclusion that PLK1 is a key mediator of plasticizer-associated prostate cancer progression. Notably, the association between PLK1 and plasticizers has not received sufficient attention. While existing research has confirmed that DBP exposure can upregulate PLK1 expression in other disease models (32), no study has systematically elucidated the key role of PLK1 in the carcinogenic effects of plasticizers in PCa.

Compared to previous studies, our research represents a significant methodological advance. Firstly, based on multi-omics data analysis, we integrated a multi-level strategy encompassing bioinformatics, machine learning algorithms, molecular docking simulations, and *in vitro* validation, which greatly enhances the systematic nature and scientific rigor of the study. Prior research often relied on database-derived epidemiological surveys and did not utilize multi-omics and multi-dimensional methods to reveal the complex interactions between multiple genes and environmental factors in PCa pathogenesis, a clear limitation of traditional approaches in this field.

This study has several limitations, which also point to clear directions for future research. First, although our *in vitro* experiments were conducted in DU145 and PC-3 cells, which represent androgen-independent prostate cancer, future studies should also include androgen-sensitive cell lines (e.g., LNCaP, 22Rv1) to fully capture tumor heterogeneity and hormonal status. Second, while this study focused on the parent plasticizer compounds (DEP, DMP, DOP), we acknowledge that phthalates undergo rapid metabolic hydrolysis *in vivo* to monoester metabolites (e.g., mono-ethyl phthalate, mono-methyl phthalate, and mono-octyl phthalate), which may possess distinct biological activities and toxicokinetic profiles. Our decision to use parent compounds was based on the compatibility with computational target prediction tools, the predominance of parent compound data in toxicogenomic databases, and the need for direct comparability with existing mechanistic studies. However, we recognize that metabolites represent the primary forms detected systemically and may be more directly relevant to human exposure scenarios. Future studies should therefore investigate whether these metabolites interact directly with PLK1 or modulate its expression through alternative mechanisms, and comparative analyses of parent compounds versus metabolites using metabolomics approaches will be essential to fully elucidate the active agents in plasticizer-associated prostate cancer progression.

Third, although the concentrations used in our *in vitro* experiments are consistent with those in mechanistic toxicology studies, they exceed typical human serum levels. To better reflect real-world exposure scenarios, future research should employ chronic, low-dose exposure models, including *in vivo* animal studies using environmentally relevant concentrations, to validate the role of PLK1 in plasticizer-induced prostate carcinogenesis under physiological conditions. Furthermore, dose–response experiments would provide critical information for assessing the translatability of our findings, and should be incorporated in future investigations.

Finally, several environmental toxicology considerations warrant further discussion. First, while this study focused on the parent plasticizer compounds (DEP, DMP, DOP), it is important to note that phthalates undergo rapid metabolic hydrolysis *in vivo*, converting to monoester metabolites that may possess distinct biological activities and toxicokinetic profiles. Future studies should investigate whether these metabolites interact directly with PLK1 or modulate its expression through alternative mechanisms. Second, the concentrations used in our *in vitro* experiments were selected based on ranges commonly employed in mechanistic toxicology studies and informed by environmentally relevant exposure levels reported in the literature. Although these concentrations exceed typical human serum levels, they are reasonable for pathway elucidation within practical experimental timeframes and are consistent with doses used in previous phthalate mechanistic studies. Nevertheless, extrapolation to real-world human exposure scenarios should be approached with caution, and future research employing lower-concentration, chronic exposure models would better recapitulate physiological conditions.

## Conclusion

In conclusion, by integrating network toxicology, multi-omics analysis, and machine learning algorithms, this study systematically reveals that common plasticizer exposure (DEP, DMP, DOP) may promote PCa initiation and progression through upregulation of PLK1. These findings have potential translational implications: PLK1 expression levels could serve as a functional biomarker for assessing individual susceptibility to plasticizer-associated prostate cancer risk in environmentally exposed populations. For instance, PLK1 expression measured in prostate biopsy specimens or liquid biopsies (e.g., circulating tumor cells or extracellular vesicles) could be integrated into risk stratification frameworks to identify high-risk individuals who may benefit from enhanced surveillance or early intervention. Furthermore, given PLK1’s druggable kinase domain, our findings support the exploration of PLK1 inhibitors as a therapeutic strategy for plasticizer-associated prostate cancers, particularly in patients with high PLK1 expression. Future studies should validate these translational applications in prospective cohort studies and preclinical models.

## Data Availability

The public datasets used in this study are available from TCGA (https://portal.gdc.cancer.gov/) and GEO (https://www.ncbi.nlm.nih.gov/geo/), with accession numbers: TCGA-PRAD, GSE21032, GSE70770, GSE116918, GSE185344, and GSM7841733.

## Supplementary material

Supplementary Figure 1 COX regression results of 18 common prognostic genes in the TCGA cohort.

Supplementary Figure 2 KM generation curves of three core genes in three cohorts.

Supplementary Figure 3 The relationship between PLK1 expression level and disease-free survival of different cancers in the TCGA pan cancer cohort.

Supplementary Figure 4 Effects of DMP on PLK1 expression levels in the DU145 cell line. *p<0.05 DMP VS Control.

Supplementary Figure 5 Validation of DMP effects in PC-3 cells. **(A)** PLK1 mRNA expression measured by qPCR in PC-3 cells after DMP treatment with or without PLK1 knockdown. **(B)** PLK1 protein expression by Western blot. **(C)** EdU proliferation assay; **(D)** Transwell invasion assay; **(E)** Wound healing migration assay. Data are mean ± SD. *p < 0.05 vs. Control; #p < 0.05 vs. DMP.
