# Supplementary figures and images for: Plasticizers and prostate cancer: unraveling the link through network toxicology and machine learning
Source: Front Oncol. 2026 Jul 9;16:1768691. doi: 10.3389/fonc.2026.1768691 (PMC13391259; doi:10.3389/fonc.2026.1768691)

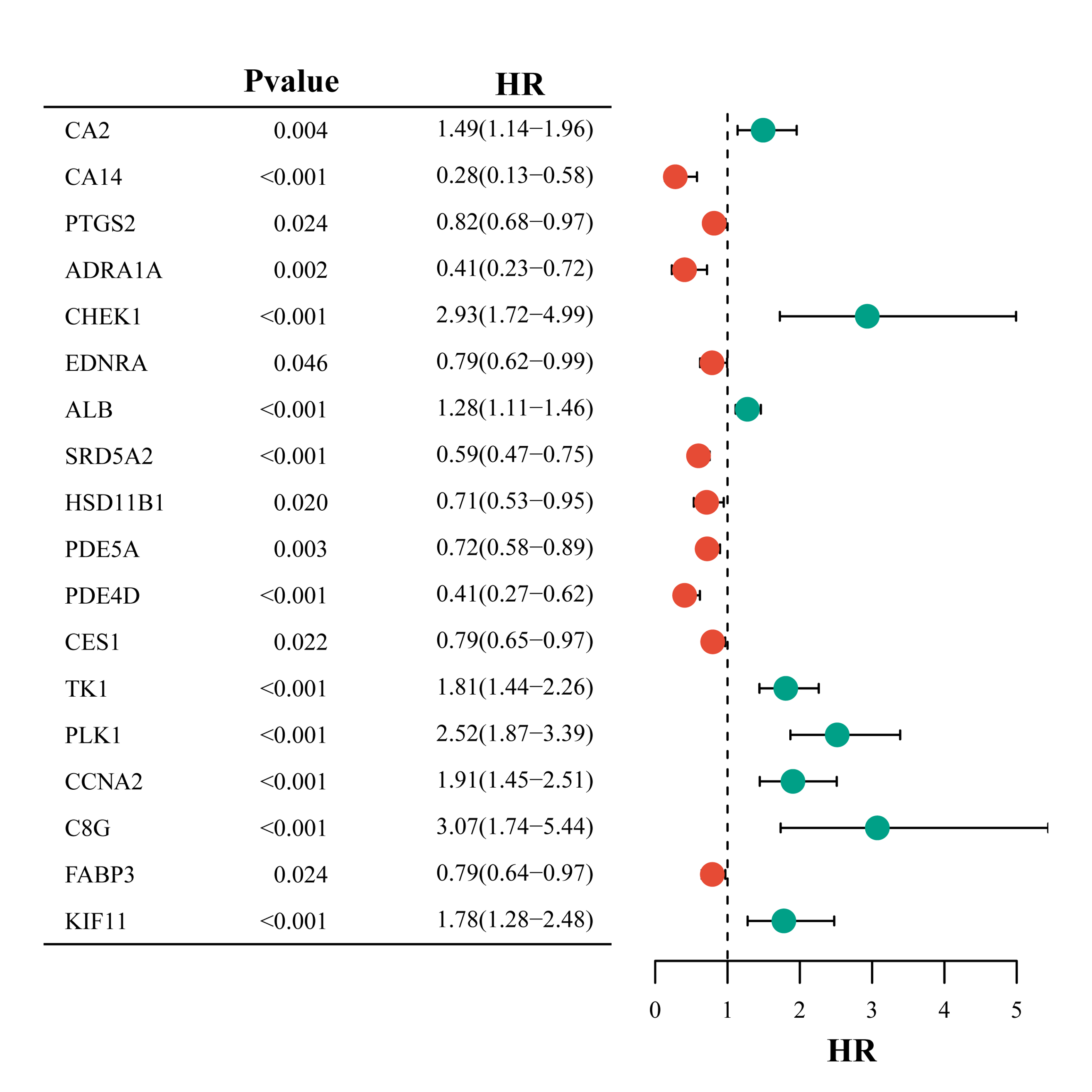

Supplement: Supplementary Figure 1 — COX regression results of 18 common prognostic genes in the TCGA cohort. [file Image1.png]

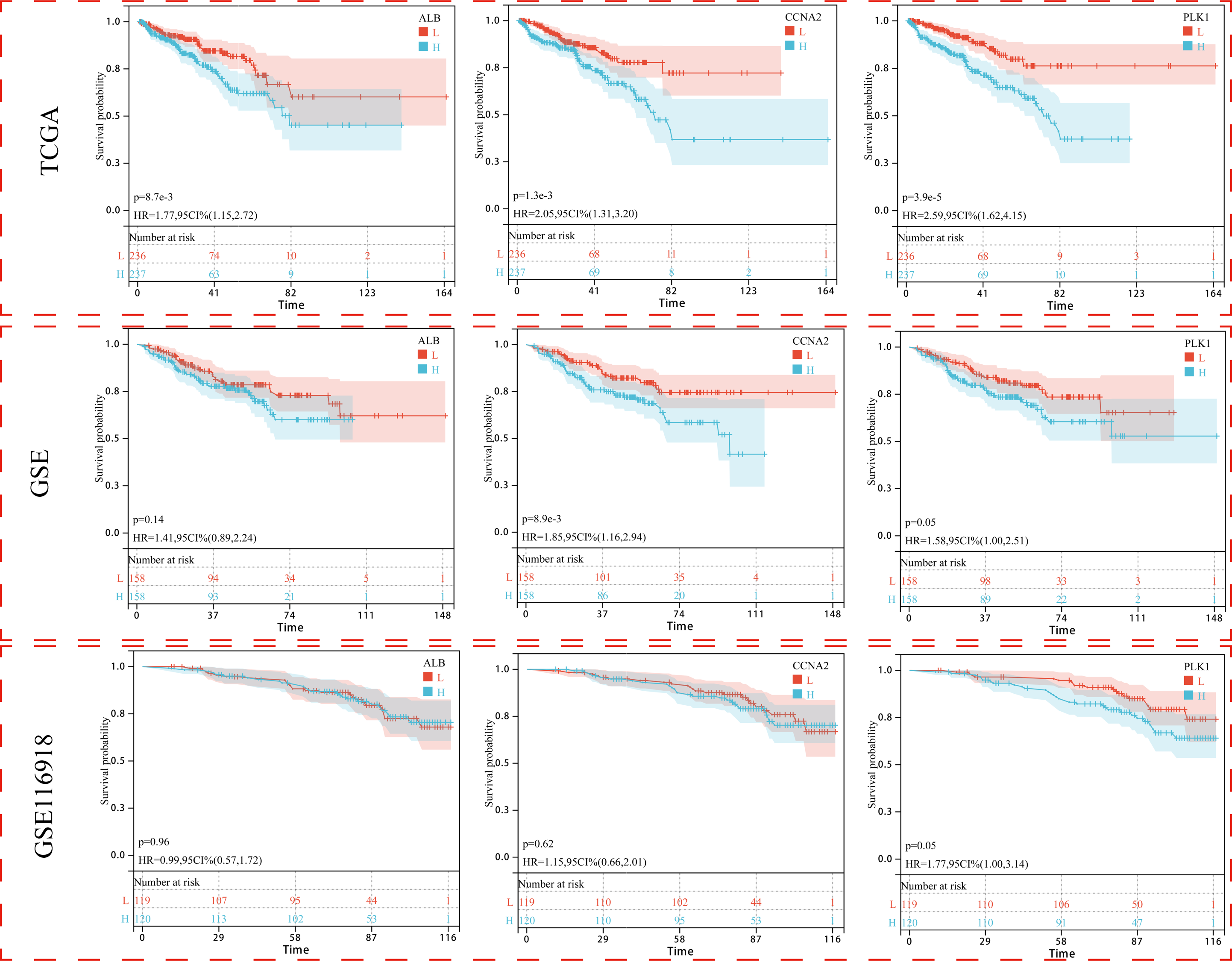

Supplement: Supplementary Figure 2 — KM generation curves of three core genes in three cohorts. [file Image2.png]

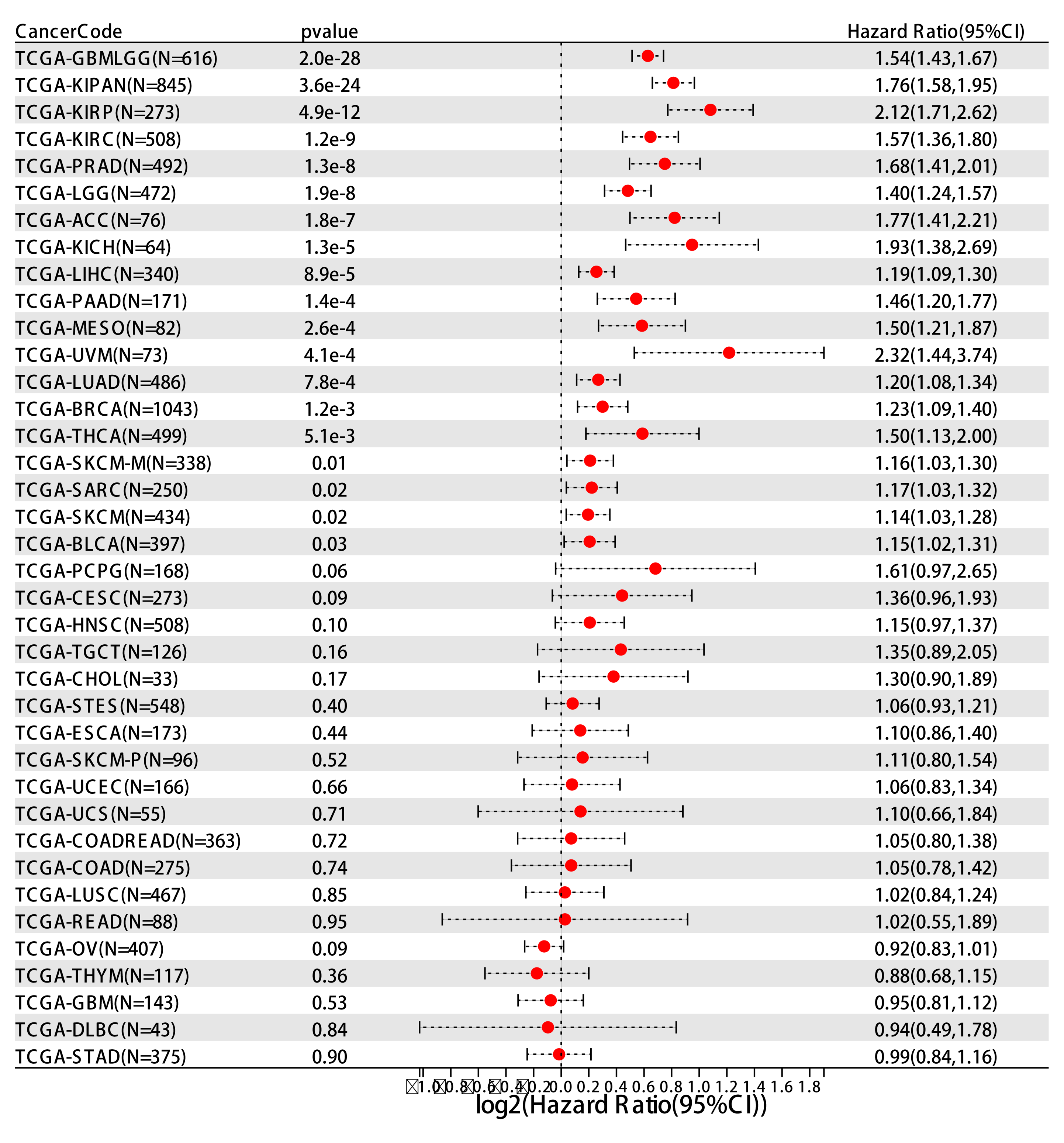

Supplement: Supplementary Figure 3 — The relationship between PLK1 expression level and disease-free survival of different cancers in the TCGA pan cancer cohort. [file Image3.png]

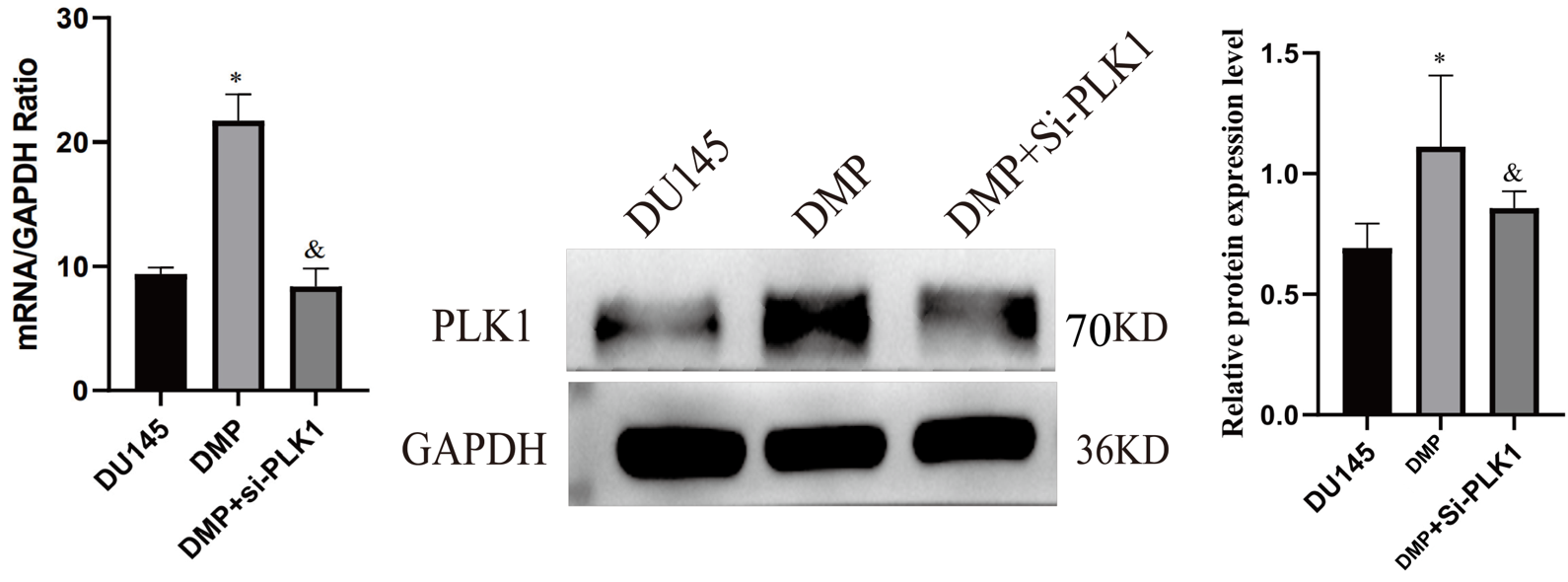

Supplement: Supplementary Figure 4 — Effects of DMP on PLK1 expression levels in the DU145 cell line. *p<0.05 DMP VS Control. [file Image4.png]

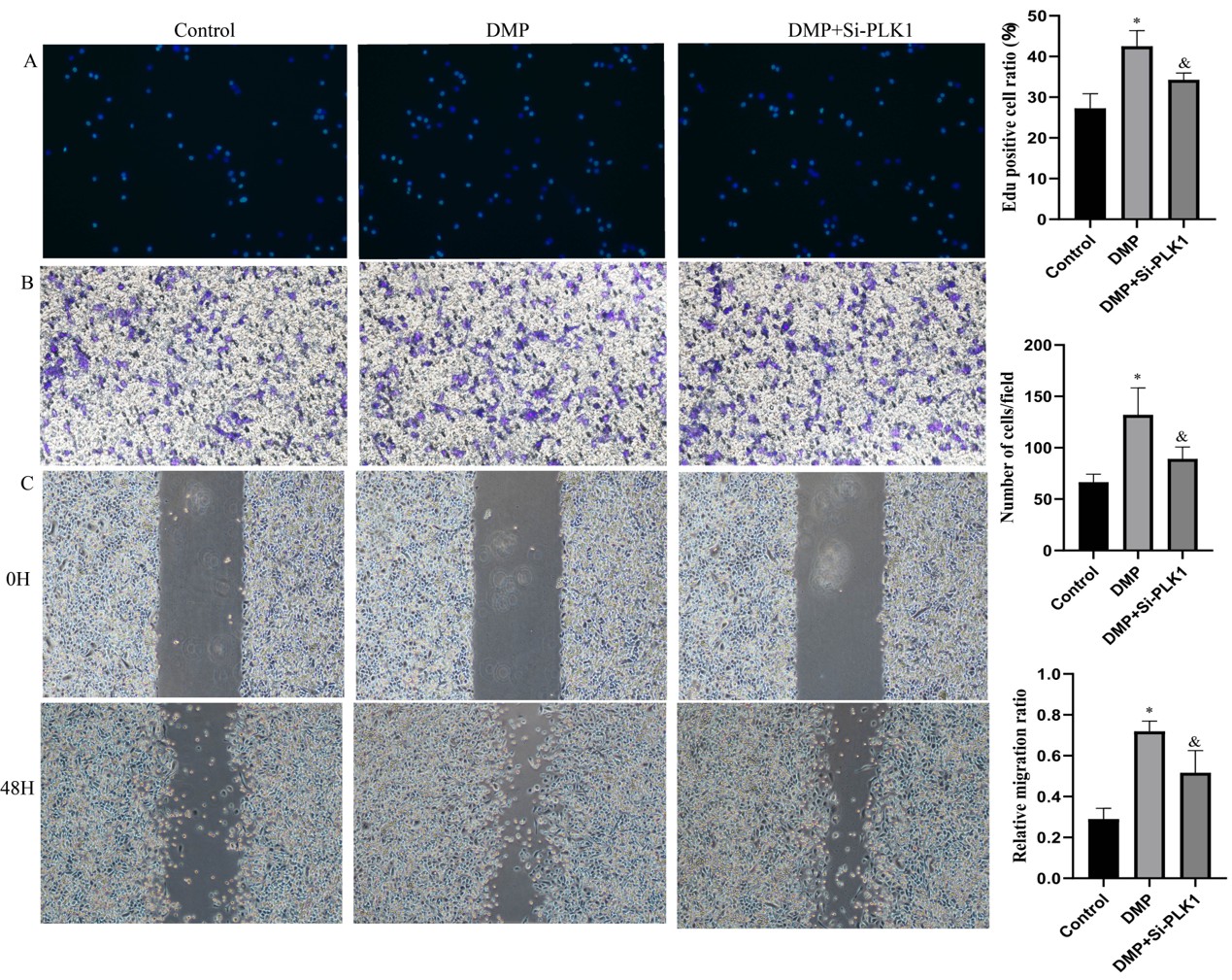

Supplement: Supplementary Figure 5 — Validation of DMP effects in PC-3 cells. (A) PLK1 mRNA expression measured by qPCR in PC-3 cells after DMP treatment with or without PLK1 knockdown. (B) PLK1 protein expression by Western blot. (C) EdU proliferation assay; (D) Transwell invasion assay; (E) Wound healing migration assay. Data are mean ± SD. *p < 0.05 vs. Control; #p < 0.05 vs. DMP. [file Image5.jpg]
